# Supplementary material for: Learning curve and surgical outcome of robotic assisted colorectal surgery with ERAS program
Source: Sci Rep. 2022 Nov 29;12:20566. doi: 10.1038/s41598-022-24665-w (PMC9709162; doi:10.1038/s41598-022-24665-w)
Supplement: Supplementary file 2 — Supplementary Information 2. [file 41598_2022_24665_MOESM2_ESM.zip › RAL Raw data-Ñ[▒K/057/HS_ñw╝╨░O▒Kñσ.pdf]

正式摘要(電子簽章：000000210046931)

W55 / W55 ☐英文標題(注意:無英文姓名-請補病人基本檔!) ☐【讀取】憑證

臺中榮民總醫院

出院病歷摘要

轉入號： 轉出號：

[redacted]

(8)流水編號	
(9)入院日期	110/01/26 (2021) 直腸外科 W55 - 262
(10)轉科日期	
(11)出院日期	110/02/09 (2021) 住院天數計 14 日

診斷

- 入院：1.Malignant tumor of descending colon,pT3N1M0,Stage IIIB
  2.Hypertension.
- 出院：1. Adenocarcinoma, descending colon, s/p robotic anterior resection on 2020/1/28
  pT3N1(IMA0/10, IMV 0/3, Region 0/16)M0,Stage IIIB
  MD, AI-, PI-, TuDp-, CRM2.7cm-, Prox:12.5cm, Dist:5.1cm, ExtNd-, pMMR,
  2.Hypertension.

主訴

- Lower abdominal cramping pain and bowel habit change with stool in thin shape for 2 months.

病史

- This is a 49-year old male with history of
  1. Hypertension.
  2. Smoking 1 PPD for 20 years.
  3. Graft surgery for burns at right leg 20 years ago.
  This time, he suffered from abdominal cramping pain for about two months with poor appetite, fatigue easily and body weight loss from 100kg to 95kg within two months. Bowel habit change was also noted from once a day to twice a week with stool in thin shape. Therefore, he came to LMD for help first, where Stool Occult Blood showed positive. He then went to Tungs' Taichung MetroHarbor Hospital where colonoscopy demonstrated descending colon tumor. Due to personal reason,he came to Dr.林's OPD for secondary opinin on 2021/01/18, where CEA=3.76,CA199=8.13, abdominal CT revealed descending colon tumor. Sigmoid colonoscopy was also arranged up to 30cm and found stool impaction. PET done on 2021/01/19 demonstrated descending colon tumor. Lung CT showed no metastasis. The patient denied fever, recent trauma, diarrhea, tarry stool, hematochezia. Stuffy nose, cough with yellish sputum were noted. Under the impression of malignant tumor of descending colon,pT3N1M0,Stage IIIB and well discussion with the patient and his family, he was admitted to our ward for robotic assisted anterior recession.

體檢發現

- CEA: 3.76

  2021/1/18 CT: D-S colon ca.
  S scopy to 30 cm, stool impaction
  1/18 abd CT: D colon ca.
  1/19 PET: D colon ca. lung CT no met.

  - General appearance :
    - easy looking , conscious clear , without cardiopulmonary distress
    - body weight : 93.7 kg , height : 168.7 cm
    - Vital signs: B.P. 116/78 mmHg , P.R. 76 /min , R.R. 18 /min , B.T. 36.3 ℃
  - HEENT :
    - Eyes: no anemic conjunctiva, no icteric sclera, normal EOM
    - ENT: no otorrhea, no rhinorrhea, no erythematous mucosa, no injected tonsil, no deformity
  - Neck: supple, freely movable, trachea midline,no deformity, no engorged jugular

- vein , no palpable thyroid or lymph node enlargement
  - Chest:
    - Inspection: Grossly normal, symmetric and fully expansion, no accessory muscle used, no operation scar, no spider angiomata, no gynecomastia
    - Palpation: no palpable mass or nodule, no subcutaneous emphysema, no tenderness
    - Ausculation: clear breathing sound
  - Heart:
    - Palpation: no thrill, no heave
    - Percussion: normal heart size
    - Ausculation: regular heart beat, normal S1, S2, no gallop, no murmur
  - Extremities: No deformity, full ranged of motion without limitation, no clubbing finger or nail cyanosis, no pitting edema, no ulcer or pigmentation, no obvious skin lesion, no varicose vein, no bone, joint or muscle tenderness

CRS CONDITION

- Karnofsky performance status: (100) Normal, no complaints, no evidence of disease
- PE of Abdomen:
- __1. Inspection: normal
- __2. Ausculation: normal
- __3. Palapation: no tenderness
- __4. Percussion: normal
- CEA: 3.76 ng/ml
- Imaging Studies:
- Colonoscope report: d-colon ca
- Figures:
  1. colon & rectum

手術日期及方法

- 手術日期及方法20210127 Robot Assisted Colectomy(L)

住院治療經過

- After admission, Robot Assisted Colectomy(L) was performed on 1/27 for malignant tumor of descending colon,pT3N1M0,Stage IIIB. Pain control PCA use was given. We let him try water and remove Foley on 1/30. No flatus at weekend was noted. KUB showed focal gaseous distension of colon, ileus should be considered. So, we hold the Bland-I order and keep try water on 2/1. After he Had stool passage once at 2/1 night, we let him try Bland-I diet on 2/2. Then, he started try Bland-II diet on 2/4 due to the tolerable intake status. Now, he can be discharge under stable condition and tolerate with Bland-II diet.

合併症

一般檢查

- 累積報告 :

DATE	TIME	ALB	TP	BIL.T	BIL.D	ALKP	AST	ALT	LDH	CHOL	TG	HDL-C	LDL	UA	NH3	AMY
1100128	0246	3.6		0.4			19	17								
1100201	0909			0.6			25	15								
1100204	0852	3.5		0.58	0.19		72	52								
1100205	1049			0.7	0.2	89	75	60								

DATE	TIME	WBC	RBC	HGB	HCT	MCV	PLT	BLST	NEUT	BAND	SEG	LYM	MON	EOS	BAS
1100128	0245	12230		14.6	43.5		249		89.5			7.7	2.7	0.0	0.1
1100201	0910	5210	3.58	12.7	37.6	105.0	218		58.1			28.8	7.5	5.0	0.6
1100204	0857	6840	3.75	13.2	38.4	102.4	247		68.3			20.0	7.5	3.9	0.3

DATE	TIME	NA	K	CL	CA	BUN	CREAT	Mg	P	CRP	ESR	PCT	LIPASE	LACTATE
1100128	0246	142	4.0		8.1	8	0.83							
1100201	0909	142	4.7	107	8.4	9	0.85							
1100204	0852	139	3.9		8.1	8	0.76							

特殊檢查

病理報告

-

- 活體組織切片病理報告　手術日期：**110/01/28**　病理號：**S1003423**

診斷醫師:陳律臻 報告日期:110/02/03
Pathologic diagnosis:
1. Descending to sigmoid colon, robotic left hemicolectomy --- Adenocarcinoma, moderately differentiated, of descending colon. Tumor invaded to mesocolic soft tissue. The bilateral cut ends and mesocolic lymph nodes (0/16) are free of tumor.
2. Colon, cut end, distal, resection --- Negative for malignancy.
3. IMA root, dissection --- Unremarkable fibrovasculoadipose tissue and lymph nodes (0/10).
4. IMV root, dissection --- Unremarkable fibrovasculoadipose tissue and lymph nodes (0/3).
Ancillary study for diagnosis:
IHC stains for MLH1, MSH2, PMS2 and MSH6 are done on section C. All above markers are preserved.
Prognostic and predictive factor:
1. Tumor size: 5x4 cm.
2. Depth of invasion: Pericolonic tissue.
3. Histologic grade: G2: Moderately differentiated.
4. Angiolymphatic invasion: Not identified.
5. Perineural invasion: Not identified.
6. Tumor deposits (discontinuous extramural extension): Not identified.
7. Macroscopic tumor perforation: Not identified.
8. Surgical Margins: a) Proximal Margin: Uninvolved by invasive carcinoma, distance of tumor from margin: 12.5 cm. b) Distal Margin: Uninvolved by invasive carcinoma, distance of tumor from margin: 5.1 cm. c) Radial or Mesenteric Margin: Uninvolved by invasive carcinoma, distance of tumor from margin: 2.7 cm.
9. Lymph node status: Uninvolved (0/29).
10. Extranodal involvement: Not identified.
11. Tumor regression grading S/P CCRT: No known presurgical therapy.
12. Type of polyp in which invasive carcinoma arose: Not identified.
13. Additional pathologic findings: Not identified.
14. Pathological TNM Stage: pT3N0(According to the eighth edition, American Joint Committee on Cancer Staging Guidelines for Tumors).
15. TNM descriptors: Not applicable.
Gross description:
The specimen consists of 1) a segment of colon, 26 cm in length and 6.5 cm in proximal cut end circumference, 6.5 cm in distal circumference, and mesocolic soft tissue. A gray tan firm annular elevated and centrally ulcerative tumor, 5x4 cm, is noted, 5.1 cm to the distal cut end. On cross section, the tumor invades to the mesocolic soft tissue with 2.7 mm to the radial margin, and serosal retraction is also noted. The both cut ends are free of tumor grossly. 2) distal cut end, a piece of tan mucosa-covered soft tissue, 2.2x2x0.8 cm. 3) IMA root, a piece of tan yellow soft tissue, 3.2x2.5x1.5 cm. 4) IMV root, a piece of tan yellow soft tissue, 4x3.5x1 cm. Representative part for section: A) proximal cut end B) distal cut end C-F) tumor G) radial margin H) non-tumor part M-N) lymph nodes J) specimen 2 K) specimen 3 L) specimen 4. Blue ink indicated serosa; yellow ink indicated radial margin.
Note:
1. The interpretation of MMR panel and recommended studies are as below: (1) No loss of nuclear expression of MMR proteins: low probability of microsatellite instability-high (MSI-H) . (2) Loss of nuclear expression of MLH1 and PMS2: testing for methylation of the MLH1 promoter and/or mutation of BRAF is indicated (the presence of a BRAF V600E mutation and/or MLH1 methylation suggests that the tumor is sporadic and germline evaluation is probably not indicated; absence of both MLH1 methylation and of BRAF V600E mutation suggests the possibility of Lynch syndrome, and sequencing and/or large deletion/duplication testing of germline MLH1 may be indicated). (3) Loss of nuclear expression of MSH2 and MSH6: high probability of Lynch syndrome (sequencing and/or large deletion/duplication testing of germline MSH2 may be indicated, and, if negative, sequencing and/or large deletion/duplication testing of germline MSH6 may be indicated). (4) Loss of nuclear expression of MSH6 only: high

- probability of Lynch syndrome (sequencing and/or large deletion/duplication testing of germline MSH6 may be indicated). (5) Loss of nuclear expression of PMS2 only: high probability of Lynch syndrome (sequencing and/or large deletion/duplication testing of germline PMS2 may be indicated). (6) Please note: There are exceptions to the above IHC interpretations. These results should not be considered in isolation, and clinical correlation with genetic counseling is recommended to assess the need for germline testing.
  2. This case has been peer reviewed by two doctors.
  #T-59461_2 #M-81403_2 2 1105 S0000F
  #T-C4560_2 #M-09183_2 0 1105 000000

其它

出院時情況

- 3.改門診治療

出院指示

藥名	劑量	劑量單位	用法	頻次	數量	數量單位	現況	天數	單位
Through tab	2	TAB	PO	QN	28	TAB	TH	14	天
Gaslan tab 40mg	1	TAB	PO	QID	56	TAB	TH	14	天
EM Voren cap 50mg	1	CAP	PO	BID	14	CAP	TH	7	天

出院計劃

主治醫師 2222H 林俊余 / 住院醫師 2353C 楊立宇

正式摘要**(**電子簽章：**000000210046931)**

W55 / W55

doctorRank:B
